# Validation of the Adolescent Health Quality of Care (AHQOC) index for mystery client studies

**DOI:** 10.1371/journal.pone.0285888

**Published:** 2023-06-15

**Authors:** Olujide Arije, Jason Madan, Tintswalo Hlungwani

**Affiliations:** 1 Institute of Public Health, Obafemi Awolowo University, Ile-Ife, Nigeria; 2 School of Public Health, University of Witwatersrand, Johannesburg, South Africa; 3 Warwick Medical School, University of Warwick, Warwick, United Kingdom; Marie Stopes International, PAKISTAN

## Abstract

The Adolescent Health Quality of Care (AHQOC) index is a tool designed to evaluate the quality of facility-based adolescent sexual and reproductive health (ASRH) services. This descriptive cross-sectional study aimed to validate the AHQOC index in 27 primary and secondary public health facilities located in a rural and an urban local government area (LGA) of Ogun State, Nigeria. To conduct the study, 12 mystery clients (MCs) were recruited and performed 144 visits to the health facilities. The MCs were young males and females who were seeking information on premarital sex, pregnancy prevention, sexually transmitted infections (STIs), and contraception. The validity, and reliability of the AHQOC index were evaluated using exploratory factor analysis, Cronbach’s Alpha, and intra-class correlation coefficient tests. The Kaiser-Meyer-Olkin test result for the initial 37-item pool was 0.7169, and the final tool retained 27 items with a Cronbach’s Alpha of 0.80. Two subscales of the index had Cronbach’s Alpha of 0.76 and 0.85. The intra-rater consistency assessed by the intra-class correlation coefficient was 0.66 (0.10–0.92) p = 0.001 for the urban LGA and 0.72 (0.37–0.91) p = 0.001 for the rural LGA. Positive and statistically significant relationships were observed between the full scales and subscales and the validity item (MC ranking of health worker on proficiency from 1 to 10). The results of this study demonstrate that the validated AHQOC index is a valuable tool for assessing the quality of ASRH services in public health facilities.

## Introduction

Evaluation of the quality of adolescent sexual and reproductive health (ASRH) services by adolescents and young people who are service users is critical to understanding their experience of care. While some authors suggest that clients may not be able to assess the appropriateness of specific technical procedures, they can evaluate other aspects of quality, such as equipment availability and the behavior of health workers [1, 2]. One helpful method for evaluating the quality of sexual and reproductive health (SRH) services for adolescents and young people is the mystery client (MC) approach, which uses trained (standardized) observers to report their experiences of service provision [3]. In this approach, the observers pose as regular users of the service in question without the service provider’s knowledge [3, 4]. This approach allows for monitoring and evaluation of the performance of healthcare providers or facilities from a user’s perspective, assuming that the service provider would provide services to such a person as they would with any other client. This effectively removes the "Hawthorne effect" that could introduce bias if the provider was aware of being observed [5]. The approach is beneficial because the findings give insight into what can be considered typical performance by service providers.

The mystery client approach has been commonly used in adolescent sexual and reproductive health services research in low, middle, and high-income countries [3, 6–8]. Both quantitative and qualitative methods have been used in MC studies [3]. Despite the widespread use of the MC methodology in adolescents and young people’s SRH research, the review by Chandra-Mouli et al. [3] showed that studies have rarely reported the process of developing and validating their study tools. In the reviewed studies, debriefing of MCs was done by filling out observation reports [9], completing a survey questionnaire [10, 11], or having reflection sessions on the MCs’ experiences [12]. Additionally, several of the reviewed studies did not elaborate on the method of debriefing used. One notable exception is the work of Castro et al. [6], who used a quality of services outcome measure based on the World Health Organization (WHO) criteria of acceptability, accessibility, effectiveness, and appropriateness to create the adolescent-friendly service (AFS) score as the sum score of 17 dichotomous items. However, most of the literature fails to include statistical validation of their study instruments in their data analysis approach. These observations indicate a gap in the statistical validation of research instruments in adolescent sexual and reproductive health (SRH) MC studies. Statistical validation helps demonstrate the reliability and other psychometric properties of research tools [13].

In our study on the quality of SRH services for adolescents and young people in Nigeria, we created a mystery client questionnaire by aligning question items obtained from previous studies thematically to the Nigerian *National Standard and Minimum Package for Adolescent Healthcare* [14]. The national standards include competencies and motivation of health workers; quality assurance and improvement; acceptable services; gatekeepers’ support; equitable and rights-based services; appropriate package and effective service; young people’s involvement; accessibility of services; and privacy and confidentiality. In this report, we discuss the validation of the Adolescent Health Quality of Care (AHQOC) tool used to assess SRH services for adolescents and young people. This study aims to establish the validity and reliability of the AHQOC Index as a tool for measuring the quality of healthcare provided to adolescents in mystery client studies by examining its internal structure. The findings of the quality assessment conducted by the mystery clients are discussed elsewhere [15].

## Methodology

### Study design, location, and sample

We carried out a descriptive cross-sectional study to evaluate the quality of adolescent sexual and reproductive health care provided in selected primary and secondary healthcare facilities in Ogun State, Southwest Nigeria. The aim of the study was to assess the quality of care in selected public primary and secondary health facilities in two local government areas (LGAs) of the study state. To achieve this, we purposively selected Abeokuta South LGA, which is predominantly urban, and Ijebu East LGA, which is predominantly rural. Thirteen out of the 16 public primary health care (PHC) facilities and two out of three public secondary facilities in Abeokuta South LGA were selected, along with 11 out of the 23 public PHC facilities and one public secondary facility in Ijebu East LGA. While all available facilities were eligible, the assessed PHC facilities were selected through systematic sampling, and the secondary facilities were selected purposively. Given the small number of the available health facilities in the study location, formal sample size calculation was not conducted. We had the goal of achieving an equivalent number of sampled health facilities in both study LGAs. However, it was necessary to assign replacement health facilities to two mystery clients for two assigned visits in the urban LGA owing to concerns that their identities might be revealed at their originally designated facilities. Consequently, this resulted in an uneven distribution of health facilities between the two study LGAs. Overall, the assessment covered a total of 27 health facilities (15 in the urban LGA and 12 in the urban LGA), comprising 24 PHC facilities and three secondary facilities.

### Item pool design

The questionnaire design was based on the critical stages recommended by King et al. [16] for developing a standardized patient questionnaire, which include obtaining preliminary information to inform tool design, writing the script, developing the questionnaire, and piloting. The review of relevant literature informed a decision to adapt of the MC scripts from the work of Boyce and Neale [4]. Also, the initial questionnaire items were derived from existing mystery client/client exit questionnaires for adolescent and young people SRH research, such as Boyce and Neale [4], and the Adolescent Client Exit Interview tool in the WHO’s Global Standard for quality healthcare service for adolescents [17]. The pool questions were then revised to align thematically with the adolescent health quality of care standards of the Nigerian *National Standard and Minimum Package for Adolescent Healthcare* [14]. The questionnaire consists of question items covering the following areas: background characteristics of the assigned mystery client (MC) case, characteristics of the visited health facility, accessibility of the health facility, acceptability of the services, equitableness and rights-based services, competency and motivation of the health workers, provision of privacy and confidentiality, appropriateness and effectiveness of services, and global assessment of the services received on the day of the visit.

### Recruitment and training of mystery clients

For this study, we recruited and trained twelve male and female mystery clients (MCs) aged between 18 and 24 years. We recruited them from two Schools of Nursing within the study location with the permission of the respective school principals. The MCs underwent a three-day training program to master scripts developed from selected scenarios. These scenarios included male/female adolescents and young people (AYP) seeking information regarding sexually transmitted infections (STI), requesting for condoms, seeking information about general family planning, and seeking counseling about pressures to have intercourse. The scripts were jointly created from the scenarios by all participants, and they typified the AYP in the study location. The script details provided allowed for standardization across the mystery clients. Biographies describing the social background, age, occupation, family details, and individual circumstances were also included along with the scripts for each scenario (see Box 1).

**Box 1. Mystery client scenarios.**

**Table:** 

**1. Requesting information regarding STI** An 18/19-year-old male wants information regarding Sexually Transmitted Infections (STIs). His motivation for coming to the clinic is that he heard of a guy who got an STI from having intercourse with a commercial sex worker.Sexual history: he is sexually active and has had intercourse with approximately five women.Contraceptive use: he has used condoms only occasionally and does not like them.Reproductive health knowledge: minimal, has heard of a few diseases. The only symptom he has heard of is itching. He has heard that AIDS can kill you and thinks that AIDS only affects prostitutes. **2. Condom request:** The youth (19/20 years old) went to the health unit for condoms because s/ he is sexually active. S/he has heard about STIs/HIV and that condoms may prevent them. The youth also heard that they are accessible at health units. However, the youth has heard many strange rumors about condoms, including the notion that condoms contain HIV or that they have holes. **3. General Family Planning:** 16/17-year-old school-going youth visits the facility to obtain information on family planning services. The youth tells the provider that s/he has never had intercourse before. A schoolmate has been her/his girlfriend/ boyfriend for six months. Currently, the youth is feeling pressured by her/his girl/boyfriend to have sexual intercourse. The youth believes that if she/he does not accept s/he will leave her/him. S/he wants to avoid pregnancy to herself/his partner but knows very little about contraceptives. The youth has only heard of the pill and has many erroneous ideas about its use and possible side effects. The youth does not dare to talk with her/his parents about the problem because they are very strict and would not appreciate this kind of relationship. **4. Young adult female feeling pressured to have intercourse:** A 16/17-year-old woman comes to the facility to obtain birth control. She tells the provider that she has never had intercourse before. It becomes apparent that she is unsure if she wants to have intercourse, but she feels pressured by her boyfriend to do so. She believes if she does not give in, he will leave her, but she does not feel ready to initiate sexual activity.Her parents are very strict; she does not want them to know anything about this.Her partner: a schoolmate, has been her boyfriend for one year.Sexual history: noneContraceptive knowledge: minimal, has only heard of the pill, and has many erroneous ideas about its use and possible side effects.

During the training, the MCs enacted scripted cases, simulated post-visit interviews, and received training on risk mitigation during clinic encounters. Additionally, the MCs were trained to assess the environment of the facilities, staff, and healthcare provider behavior, effectiveness of communication, respect and preservation of dignity, and emotional support provided. The MCs were required not to give the healthcare provider additional information outside the scripts or give information that was not prompted.

After completing the training, the mystery clients piloted their designated scenarios in healthcare facilities that were not part of the study. This involved evaluating the face validity of the scenarios and the debriefing tools used by the mystery clients. In the pilot study, we assessed the feasibility of administering the AHQOC Index to mystery clients, the time required to complete the assessment, and any issues with the clarity of the items. Based on the results of the pilot study, we made minor revisions to the AHQOC Index to ensure that it was feasible for use in the main study.

### Data collection

Data was collected from July to August 2021, based on a schedule that assigned each of the 12 MCs to one health facility per day. After each visit, each MC was required to hold a debriefing session with a supervisor, during which an MC debriefing questionnaire was administered. The MCs were divided into two groups of six, and each team was assigned to one of the Local Government Areas (LGAs). Each MC visited all the selected health facilities in their assigned LGA. However, the schedule prevented more than one MC from visiting the same health facility on any given day. As stated earlier, we had to introduce replacement health facilities on two occasions. The data were collected and managed using REDCap electronic data capture tools hosted at the University of Witwatersrand [18, 19]. Qualitative data was also collected in this research using an online survey containing open-ended questions which the MCs were required to complete after each clinic visit. Findings from the qualitative data are part of a separate report [15].

### Measures and outcomes

The main outcome of interest in this research is the Adolescent Health Quality of Care (AHQOC) index, derived from a set of questions grouped into six thematic areas. These include:

- Three items on Accessibility, which assess if young people in the catchment area of the health facility were aware of the services it provides, found the health facility easy to reach and obtain services from it.
- Nine items on Acceptable services, assessing whether young people found the environment, setting, and procedures of health facilities appealing and acceptable.
- Three items on Equitable & Rights-Based Services, assessing if young people who visit health service delivery facilities were treated with respect, dignity, and in an equitable manner irrespective of their health, socio-demographic, or political status.
- Four items on Competencies and Motivation of Health Workers, assessing if the service providers were skilled and motivated to provide health services to young people in an adolescent/youth-friendly manner.
- Four items on Privacy and Confidentiality, assessing if the service providers were sensitive to the needs of young people and maintained their privacy and confidentiality in service provision.
- Eight items on Appropriate Package & Effective Services, assessing if the services provided by health facilities to young people were evidence-informed, effective, and in line with the nationally defined package.

A series of general questions tagged "Global Assessment" were included, comprising eight items, such as the cost of services, general perception about the health worker, time spent to obtain services, and willingness to recommend the facility’s services to a peer. Most of the items in the tool elicited responses of either ’Yes’ or ’No’, indicating the presence or absence of the phenomenon of interest. A score of 1 was assigned to ’Yes’, and 0 to ’No’. At times, the responses given did not strictly follow the binary ’Yes’ or ’No’ format. Instead, they appropriately corresponded with the question asked but with dichotomous responses. The items that did not fit into a dichotomous response were the items *"What did you think about the cost*?*"* that had the options of Expensive/Affordable/No cost, scored 1/2/3 respectively; "*Was the inside (and separately for outside) of the facility clean*?*"* with options of Unclean/Somewhat clean and Very clean, scored 1/2/3 respectively; and *Time spent before being attended to (waiting time)* categorized into >60 minutes/31-60 minutes/16-30 minutes/≤15 minutes, scored as 1/2/3/4 respectively (see S1 File for the final measure and scoring system of the AHQOC index). The categorization of time spent was done during data analysis. The final AHQOC index was derived by summation of responses to question items (see S1 File).

### Statistical analysis

We conducted a descriptive univariate analysis to investigate the background characteristics of both mystery clients and health facilities. Our analysis also included an examination of the distribution of the items of the final AHQOC index to identify potential ceiling and floor effects. A ceiling effect occurs when a large number of participants receive the highest possible score, indicating that the item is too easy and lacks sensitivity in detecting differences among participants. Conversely, a floor effect occurs when a large number of participants receive the lowest possible score, indicating that the item is too difficult and lacks sensitivity in detecting differences among participants. We also conducted a normality test for skewness and kurtosis to assess the normality of the distribution of the AHQOC index.

#### Exploratory factor analysis

To validate the AHQOC index, we used exploratory factor analysis (EFA) with the principal component analysis (PCA) extraction method and varimax (orthogonal) rotation. We also conducted a Kaiser-Meyer-Olkin (KMO) test to measure the adequacy of the question items for factor analysis. PCA was chosen as the most common method of EFA, aiming to account for as much variance as possible in the observed variables. The varimax rotation method was chosen to obtain a simpler and more interpretable factor structure by maximizing the variance of the squared loadings of each variable on a single factor while minimizing the sum of the squared loadings on all other factors. We acknowledge that PCA may overestimate the total variance explained (TVE), loadings, and commonalities, which could lead to an overestimation of the number of factors retained. However, we selected PCA for its ability to produce uncorrelated factors that can be more easily interpreted. Furthermore, we used a communality cutoff of 0.3 to ensure that each variable was adequately represented by the retained factors. Varimax rotation was used to produce a simpler factor structure that was more interpretable. The factors obtained were uncorrelated, and the loadings of each variable on a single factor were maximized while minimizing the sum of the squared loadings on all other factors. This method helped improve the clarity of the factor structure and facilitate the interpretation of the factors. The combination of PCA and varimax rotation allowed for the identification of factors that accounted for a significant proportion of the variance in the data, while also ensuring a simpler and more interpretable factor structure.

#### Reliability analysis

We assessed the internal consistency of the retained items using Cronbach’s Alpha to test the reliability of the full scale and the subscales. Being satisfied with the reliability characteristics of the scale, we created the AHQOC index by summing the scores of all the retained items separately for the full scale and those subscales whose Cronbach’s Alphas were 0.7 or higher.

#### Intra-class correlation

As the final step of index reliability analysis, we conducted an intra-class correlation (ICC) for the scale. We considered this necessary because of the data structure, which required all the MCs to visit all the health facilities in their LGA, presenting the particular MC case assigned to them each time. This means that each of the six MCs rated each health facility in each LGA (the only occasions this did not happen was for the replacement health facilities assigned as discussed earlier). The ICC helps to assess the consistency of the assessment across the MCs and health facilities. The ICC estimates correlations between individual measurements and between average measurements made on the same target [20]. We estimated the ICC in a two-way mixed-effect model since the same set of MCs visited each health facility in each study LGA. Koo and Li [21] recommend that ICC between 0.5 and 0.75 represent moderate reliability, while between 0.75 and 0.9 is good reliability, and greater than 0.9 excellent reliability. The ICC was reported differently for the two LGAs since different groups of MCs conducted visits in the two locations.

#### Concurrent and divergent validity of the AHQOC index and subscales

We used a linear panel-data regression model that fits random-effects models using the generalized least square (GLS) estimator and a robust estimation of variance to investigate the relationship between the total scale and each subscale to a variable that ranked the health worker encountered on a scale of 1–10. A panel data structure was used because each health facility had multiple AHQOC scores corresponding to each MC visit to the facility. Each regression model was adjusted for the age and sex of the MC, the location of the health facility, the level of care of the health facility, and the sex of the health worker encountered as follows:

$$\;Quality_{ijkt}\;=\;\alpha \;+\;\beta^{*}\;Provider_{it}\;+\;\gamma^{*}MC_{jt}\;+\;\delta^{*}\;Facility_{kt}\;+\;\epsilon_{ijkt}$$

Here, *Provider*_*i*_ is a vector of provider characteristics, including the sex of the provider and a proficiency ranking on the scale of 1–10 assigned by the MC to the provider. *MC*_*j*_ is a vector of the MC characteristics, including sex, age, and MC scenario, while *Facility*_*k*_ is a vector of health facility characteristics, including facility location and care level (primary or secondary), at each MC visit. ϵ is an error term. The health facility unique identifier was the panel variable. The statistical analyses were conducted using Stata/SE version 15 [22]. The relevant data for this study is in (see S1 Data).

### Ethical approval and consent to participate

We obtained ethical approval from the Human Research Ethics Committee of the Ogun State Primary Health Care Development Board (OGHECADEB) Ethics Committee (#OGPHC/021/008) and the University of the Witwatersrand (#M210315). The MCs who participated in the study were between the ages of 18 and 24 and were required to provide written informed consent before their participation. They were also compensated with a standard field allowance for research assistants, transportation allowance, and communication allowance during the fieldwork.

Ensuring the privacy of the service providers being assessed is of utmost importance. However, some literature has raised concerns about whether there is a breach of privacy in the MC approach, as the providers are unaware of being assessed [23]. In order to address this concern, the Medical Officer of Health in each LGA was informed about the research one week before data collection commenced. They were requested to broadcast the information on the central communication platform for all facility heads in the LGA regarding an intended MC survey within the LGA, without specifying which health facilities would be visited or when the visits would be conducted. Additionally, to mitigate any potential risks to the MCs, risk mitigation training was included in their training, as they were exposed to the risk of requesting physical examinations as part of the consultation with service providers. The MCs were also trained to terminate such clinic encounters.

## Results

This study included 144 MC visits across 27 health facilities, with 15 facilities in the urban LGA and 12 in the rural LGA. The ages of the MC scenarios ranged from 16 to 20 years, and the gender of the MC was balanced equally between males and females (Table 1). In 88.2% of the visits, the primary health worker encountered was female, and 93.1% of the visits took place in a PHC facility.

**Table 1:** Background characteristics of mystery client and health facilities (by frequency of mystery client visits).

Background characteristics	No.	%
**Scenario enacted**		
Information regarding STIs	24	16.7
Condom request	48	33.3
Information regarding contraceptives	48	33.3
Counseling regarding premarital intercourse	24	16.7
**Age of mystery client (years)**		
16	23	16
17	6	4.2
18	31	21.5
19	60	41.7
20	24	16.7
**Sex of mystery client**		
Male	72	50
Female	72	50
**Local Government Area**		
Abeokuta South	72	50
Ijebu East	72	50
**Level of facility**		
PHC Facility	134	93.1
Secondary Health facility	10	6.9
**Sex of health worker encountered**		
Male	17	11.8
Female	127	88.2
Total	144	100

**Fig 1:**
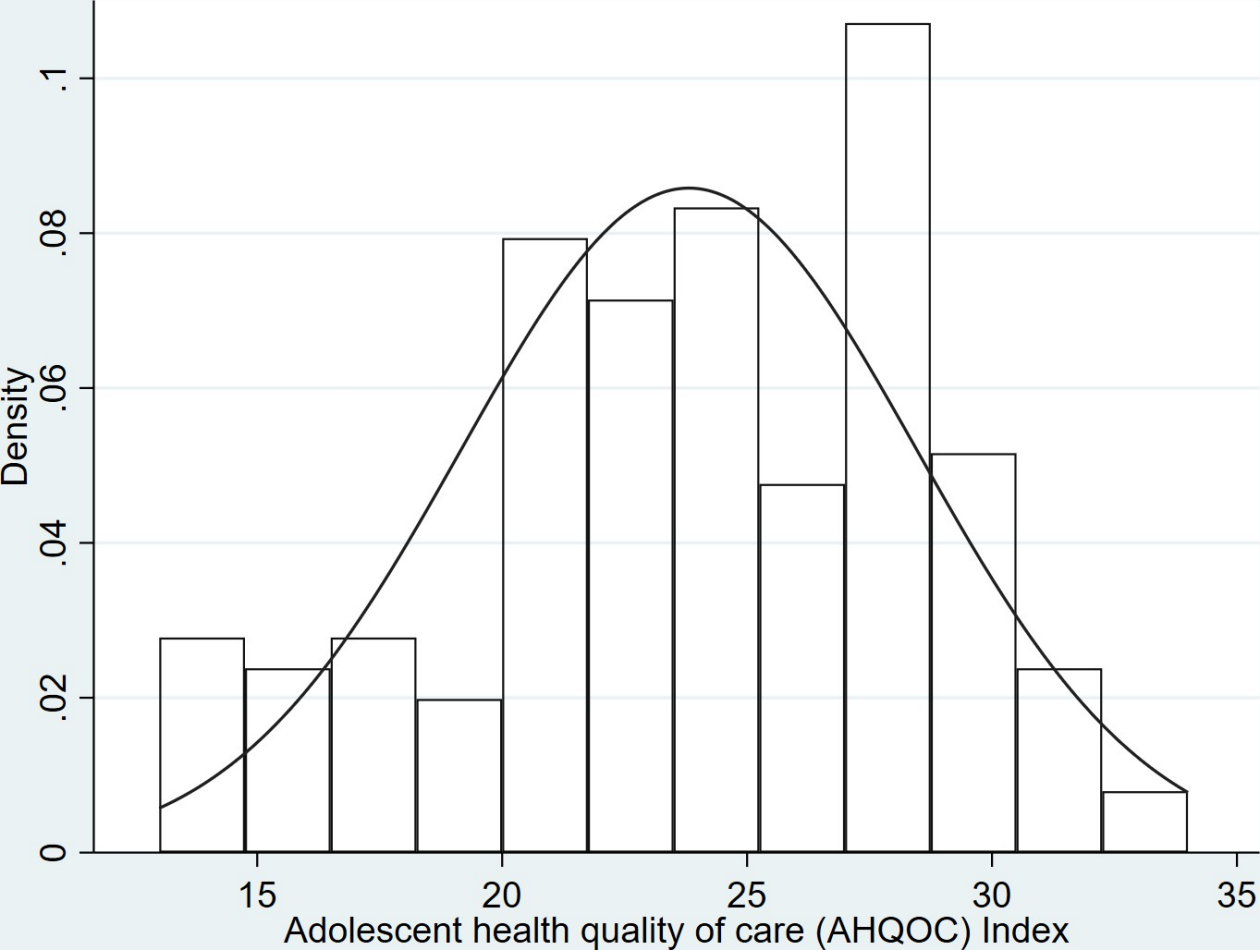
Histogram and normal density plot of AHQOC index.

From the 144 observations, the mean and standard deviation for the final AHQOC index were 23.8±4.7. The lowest score obtained was 13 (in 0.7% of cases), and the highest was 34 (also obtained in 0.7% of cases), giving a range of 21. We also examined individual index items for ceiling and floor effects. Two items had more than 80% of cases of "yes" (or corresponding response), and another two had more than 80% of cases of "no" (or corresponding response) [15]. We decided to retain these items in our index based on the findings from the exploratory factor analysis, the importance of the concepts the items represented, and their relevance to assessing SRH quality of care. The test for normality for the final AHQOC index had a skewness value of 0.0837 and a kurtosis value of 0.2050 (joint-adjusted χ² = 4.68, p = 0.0964). These findings suggest that the AHQOC index was not significantly deviated from normality, although the skewness value suggested that the distribution may be slightly skewed to the right, as may be seen in Fig 1.

### Factor analysis and scale development

We conducted exploratory factor analysis with an initial 37-item pool. The Kaiser-Meyer-Olkin (KMO) test result was 0.72 for the 37 items, indicating that our sample was adequate for factor analysis. The factor analysis produced six factors with eigenvalues of one or more. Of the 37 quality of care items, the five items with a factor loading of less than 0.3 for all six retained factors were eliminated from the varimax (orthogonal) rotated solution. Also, another five items that loaded on more than one factor after removing the factor loading less than 0.3 were eliminated. The final factor structure retained 27 items (Table 2).

**Table 2:** Adolescent health quality of care index factor analysis results*.

Category	Quality of care items	Factor1	Factor2	Factor3	Factor4	Factor5	Factor6
**Included items**							
Appropriate package & effective services	Were you counseled on any contraceptive methods?	0.36					
Appropriate package & effective services	Were you counseled on pregnancy prevention?	0.43					
Appropriate package & effective services	Did you feel the provider had adequate time for you during consultation?	0.60					
Appropriate package & effective services	Did the provider give you an opportunity to ask questions?	0.60					
Global assessment scale	Did the HW appear knowledgeable about the case you presented	0.56					
Global assessment scale	Will you recommend this facility to any of your colleague youth?	0.61					
Global assessment scale	Did he/she explain to you adequately	0.79					
Global assessment scale	In general, how did you find the counseling?	0.81					
Global assessment scale	Did he/she address your worries seriously	0.83					
Privacy and confidentiality	Were you assured of confidentiality?		0.61				
Privacy and confidentiality	Were you counseled in a place where visual privacy was guaranteed?		0.65				
Privacy and confidentiality	Were you counseled in a place where auditory privacy was guaranteed?		0.79				
Competencies & motivation of health workers	Was your medical history taken?			0.39			
Competencies & motivation of health workers	Was your sexual/reproductive history taken?			0.59			
Competencies & motivation of health workers	Was your social record taken?			0.71			
Global assessment scale	What did you think about the cost?				-0.30		
Acceptable services	Was the inside of the facility clean?				0.39		
Accessibility	Were there any directional signs within the facility?				0.40		
Accessibility	Did you find the facility easily?				0.55		
Accessibility	Were there any directional signs outside the facility?				0.63		
Appropriate package & effective services	Did the provider talk about HIV/AIDS with you?					-0.41	
Acceptable services	How did you feel about the waiting time?					0.54	
Global assessment scale	Time spent					0.64	
Acceptable services	Were there posters on STDs and other SRH issues in the facility?						0.31
Acceptable services	Was there a separate waiting room for adolescents?						0.35
Acceptable services	Was the outside of the facility clean?						0.36
Competencies and motivation of health workers	Were you asked about previous contraceptive use experience?						0.36
**Excluded items: Double factor loading**							
Acceptable services	How did you find the environment welcoming?	0.33	0.34				
Equitable & rights-based services	How would you judge the attitude of the health workers that attended to you	0.57					-0.46
Equitable & rights-based services	How would you judge the attitude of the record staff at registration point?	0.48					-0.55
Appropriate package & effective services	Were you counseled on sexual abstinence?			0.40	0.34		
Appropriate package & effective services	Were you counseled on condom specifically?	0.31		0.34			
**Excluded items: No factor loading**							
Acceptable services	Did you find any poster stating the rights of the client?						
Privacy and confidentiality	Did the provider offer to physically examine you?						
Appropriate package & effective services	Were you told where to go in case of complications?						
Appropriate package & effective services	Were you given any educational materials to read?						
Global assessment scale	Gave you a specific date to return						

* Factor loadings represent rotated factor structure, using varimax rotation

The first factor corresponded with a combination of items from "Appropriate package & effective services" and "Global assessment" and included nine items. The second factor corresponded with three items from the "Privacy and confidentiality" question items. The third factor corresponded with three items under "Competencies and motivation of health workers." The fourth to sixth factors corresponded with items from multiple categories, hence no clear-cut grouping concept was assigned. The ten items excluded were in the category of "Appropriate package & effective services" (four items), "Acceptable services" (two items), "Equitable & rights-based services" (two items), and one each from the "Privacy and confidentiality" and "Global assessment" categories. Only "Equitable & rights-based services" did not have at least one item represented in the final tool.

### Scale and subscale reliability and validity

The Cronbach’s Alpha coefficient for the 27-item scale was 0.80, indicating good internal consistency. We further examined whether the factors could be treated as individual subscales. The first factor had a Cronbach’s Alpha of 0.85, the second had 0.76, but the remaining four factors had values ranging from 0.42–0.59. Consequently, only the first two subscales were used as standalone subscales in this study. Sum scores were calculated for the full 27-item scale (AHQOC: full). We named the 9-item scale that loaded on the first factor and corresponded to the “Appropriate package & effective services” and “Global assessment” categories as AHQOC: effectiveness. Additionally, we named the 3-item scale that loaded on the second factor and corresponded to the “Privacy and confidentiality” category as AHQOC: privacy.

**Fig 2:**
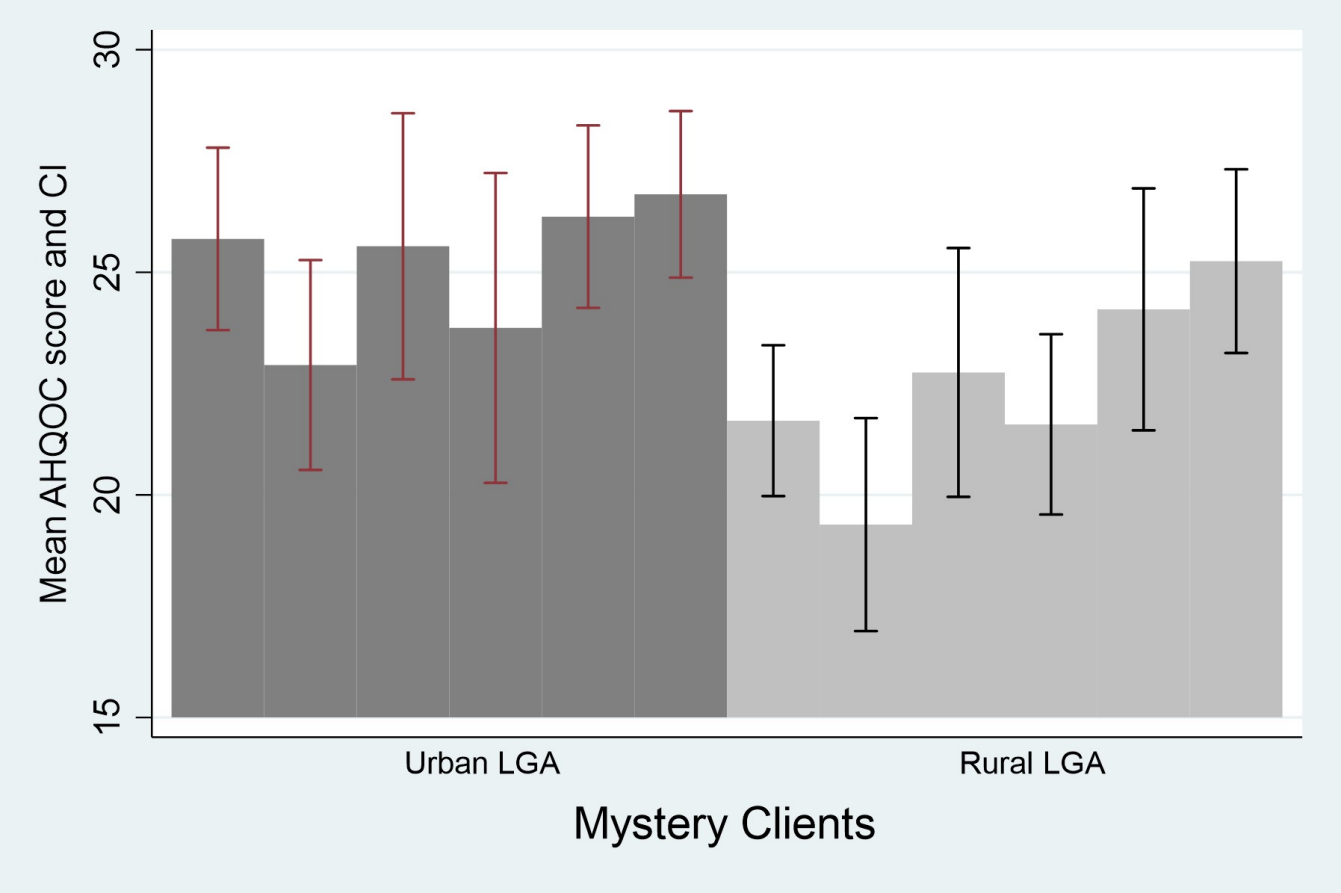
Mean score and standard deviation of AHQOC index for each mystery clients in the rural and urban LGA.

**Fig 3:**
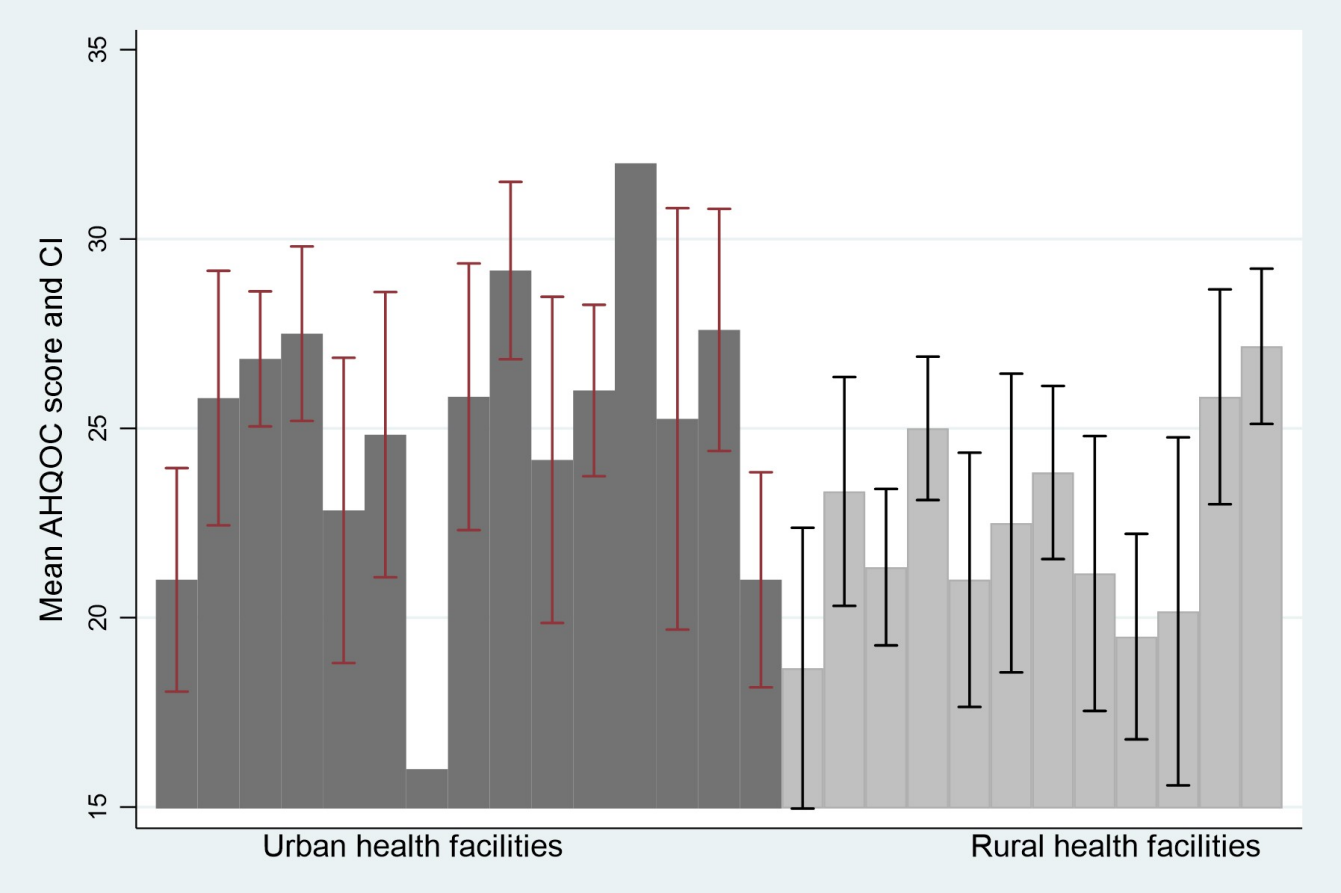
Mean score and standard deviation of AHQOC index for each health facility in the rural and urban LGA.

To assess the intra-rater consistency for the 27-item AHQOC index of the health facilities in the predominantly urban LGA, we calculated the intra-class correlation (ICC) coefficient. The ICC coefficient was 0.66 (0.10–0.92) p = 0.001 for average measures, and 0.72 (0.37–0.91) p = 0.001 for the predominantly rural LGA. As an additional assessment of interclass correlation, Fig 2 shows a comparison of the mean AHQOC score for each MC in the two study LGAs. Fig 3 shows a comparison of the mean AHQOC score for each health facility in the two LGAs. The AHQOC mean scores were generally higher in the urban LGA than in the rural LGA. Importantly, there was consistency in the scores among MCs and among health facilities in the same LGA.

We conducted panel-data regression models to investigate the relationship between the total scale and each subscale, and the ranking of health workers by the mystery clients (MCs). At the end of each interview, MCs were asked to rank the performance of the health worker they interacted with on a scale of 1–10, where 10 indicated high-quality performance. Each regression model was adjusted for provider, facility, and MC characteristics.

Our findings revealed that female MC visits had lower AHQOC index rating, and the AHQOC index reduced with increasing age of MC. Additionally, visits with female health workers resulted in lower AHQOC index on average for the full scale and the two subscales. The relationships between the full scales and subscales, and the validity item (ranking of health worker) were as expected, where a higher quality of care score was positively associated with a higher ranking of health worker. Specifically, for AHQOC: full, the β was 1.8 (1.5–2.1), p<0.001; for effectiveness, the β was 1.2 (1.1–1.3), p<0.001; and for privacy, the β was 0.2 (0.1–0.3), p<0.001 (Table 3).

**Table 3:** Association between quality of care and ranking of health care workers controlling for selected mystery client, health care provider, and health facility characteristics.

Selected characteristics	AHQOC full	p	AHQOC effectiveness	p	AHQOC privacy	p
MC ranking of health worker*	1.8 (1.5–2.1)	<0.001	1.2 (1.1–1.3)	<0.001	0.2 (0.1–0.3)	<0.001
**Scenario enacted**						
Requesting Information about STIs						
Condom request	0.5 (-1.0–2.0)	0.49	0.1 (-0.8–0.8)	0.96	0.5 (-0.13–1.08)	0.13
Information regarding contraceptives	0.2 (-1.2–1.67)	0.76	0.8 (0.1–1.6)	0.03	-0.01 (-0.6–0.6)	0.97
Counseling regarding premarital intercourse	3.0 (1.0–5.0)	<0.001	1.4 (0.4–2.5)	0.01	0.7 (-0.2–1.5)	0.11
**Age of MC**	-0.1 (-0.7–0.5)	0.78	-0.5 (-0.8–0.2)	<0.001	-0.1 (-0.3–0.1)	0.41
**Sex of MC**						
Male						
Female	-0.5 (-1.7–0.7)	0.43	-0.5 (-1.2–0.1)	0.12	-0.1 (-0.6–0.4)	0.71
**Locality of facility**						
Predominantly urban LGA						
Predominantly rural LGA	-0.4 (-1.8–1.0)	0.59	0.2 (-0.4–0.8)	0.55	0.0 (-0.5–0.5)	0.90
**Level of facility**						
Primary health facility						
Secondary health facility	0.6 (-1.5–2.8)	0.56	-0.5 (-1.4–0.5)	0.36	0.6 (-0.2–1.3)	0.15
**Sex of health worker**						
Male						
Female	-0.8 (-2.3–0. 8)	0.33	-0.6 (-1.4–0.2)	0.12	-0.5 (-1.1–0.1)	0.09
**Constant**	14.9 (3. 1–26.4)	0.01	8.6 (2.6–14.7)	0.01	2.1 (-2.6–6.8)	0.38

MC: Mystery client

*Ranking of health worker encountered by MC on the scale of 1 to 10 for proficiency

## Discussion

To the best of our knowledge, our study is the first MC study in ASRH research to report the internal consistency of the quantitative tool used [3]. Despite beginning with 37 items, we excluded 10 from the final tool based on multiple or no factor loading loaded at a communality cutoff of 0.3 after varimax rotation. Beaver et al. [24] suggested cutoff values between 0.25 and 4 as acceptable. Since Exploratory Factor Analysis (EFA) only investigates latent traits (i.e., factors) within the constructs, it is not suitable for validating factor models. The factor structures we have obtained will need validation in a future study using confirmatory factor analysis (CFA) and a different, perhaps larger set of data [25]. However, the tool developed in this study is still a valuable complement to the *Nigerian National Standard and Minimum Package for Adolescent Healthcare* [14] from which we formed the question items and used components of its national standards.

Not all factor loadings directly translated to meaningful and unique categories based on the original categorization of the questionnaire items. Nevertheless, our findings in this study demonstrate that quality of care is not a monolithic construct but has identifiable sub-constructs. The two subscales that show reliability are AHQOC: effectiveness and AHQOC: privacy, while the full scale is AHQOC: full. The full scale and the two subscales had Cronbach’s Alpha >0.7, indicating good internal consistency. Since privacy and confidentiality are crucial aspects of what drives the utilization of health services among AYP [26, 27], it is advantageous to measure it validly as a separate construct. Given that the evidence for the other subscales is weak in this study, perhaps future studies may provide further insight into their usefulness as subscales.

A key strength of the MC methodology is its ability to standardize cases presented to service providers, although this strength is also determined by the performance variation of the MCs [28]. To achieve a common understanding of what each measurement criterion meant, Boyce and Neale [4] identified the need to define all concepts and measurement criteria clearly, along with clear instructions to MCs. This should be done during training to ensure the reliability of the measure through standardization. Nalwadda et al. [29] further recommend selecting competent MCs with good communication skills and rigorous pre-data collection training to strengthen MC studies. It’s also worth noting that problems arising from variation in the performance of the MC might have been minimized through repetition in which multiple MCs present the same case to the same set of service providers [28]. We adopted this approach, in which multiple mystery clients visited the same sets of health facilities, allowing for replication to reduce measurement error due to rater biases. This was designed to strengthen the validity of the measure of quality, along with the standardization of MCs during training.

The intra-class correlation coefficient provides a measure of rater agreement or consistency. The ICC coefficient of 0.6–0.7 in this study indicated moderate reliability [21]. However, their wide confidence intervals may be due to the MC presenting different scenarios. This is likely the case, as seen in the scenarios having differential quality ratings from our regression model. The explanation may be that MCs rated similar health facilities but not exactly the same services, i.e., the same facility may offer different quality of care for different scenarios. Another possibility is variation in the care provided by a health worker in cases where the same health worker was seen by multiple MCs. We did not account for this possible variation as the MCs did not obtain personal information that would allow unique identification of each health worker because it could arouse suspicions. Intra-class correlation coefficient should be very high, the confidence interval very narrow, and the mean score not statistically significantly different across the MCs if they all presented the same cases during all their visits and were properly standardized.

There were no significant floor or ceiling effects observed for the final AHQOC index, as less than 1% of cases obtained either the lowest or highest scores, and the range of scores indicated a wide enough range of responses. Additionally, normality test results showed that the distribution of AHQOC index did not significantly deviate from normality. Overall, the absence of ceiling and floor effects and the wide range of scores make AHQOC index a good variable to use in statistical analyses.

This study found that the AHQOC index rating decreased with the increasing age of the MC, and female MC visits had a lower AHQOC index rating. Additionally, the study revealed that visits with female health workers resulted in a lower AHQOC index on average for the full scale and the two subscales. The study’s findings indicate that there are differences in the quality of care provided by health workers based on both the health worker’s gender and the MC’s gender. However, it is important to note that the quality of care is a complex and multifaceted concept that can be influenced by various factors, which could be individual, interpersonal, community or societal [30]. The study also found that a higher quality of care score was positively associated with a higher ranking of health workers, indicating that the AHQOC Index can be a valuable tool for assessing the quality of care provided by health workers. Furthermore, the similarities in the findings for the full scale and the subscales confirm the validity and reliability of the AHQOC index.

While the study provided valuable insights, it is worth noting that the AHQOC Index may not have captured all relevant aspects of adolescent health quality of care, and the use of mystery clients may not accurately reflect the experiences of real adolescents seeking healthcare services. Nonetheless, the study’s primary contribution is the validation of the AHQOC Index for use in mystery client studies in Nigeria. This fills a gap in the literature and advances our understanding of ASRH. A similar tool to the AHQOC index is the Adolescent Hospital Quality of Care Survey (AHQOCS), which evaluates the quality of care provided to adolescents in hospitals [31]. The survey consists of 31 items divided into three domains: communication and respect, pain management, and discharge planning. The AHQOCS demonstrated good content validity and test-retest reliability with a coefficient of 0.83, which is similar to the finding of our study with a Cronbach’s alpha of 0.80. The Adolescent Health Counseling Quality Questionnaire (AHCQQ), which assesses the quality of health counseling provided to adolescents, also showed good internal consistency with a Cronbach’s alpha of 0.84. The questionnaire consists of 16 items divided into four domains: building rapport, assessing behaviors, providing education, and promoting change. Additionally, the Young Adult Health Care Survey (YAHCS) evaluates the provision of preventive health services for young adults aged 18 to 26 and demonstrated good internal consistency with a Cronbach’s alpha of 0.88 and test-retest reliability with a coefficient of 0.85 [32]. The questionnaire consists of 16 items divided into four domains: building rapport, assessing behaviors, providing education, and promoting change. The content validity of the YAHCS was evaluated through expert reviews and consensus conferences. While these tools assess relevant domains of adolescent healthcare, they have a limitation in their use for comparisons with our study since none of them use the mystery client methodology. We did not find any previous mystery client studies on adolescent sexual and reproductive health that reported the psychometric properties of their tool, which we could compare our findings with.

This study has a few limitations. Firstly, there were only 144 observations in this study based on 12 MC visits by each of the 12 MCs across the selected health facilities. Perhaps the confidence interval of the ICC would be much narrower, hence more precisely estimated, with a larger sample size. Secondly, it is impossible to simulate a complete clinic experience both for practical and ethical reasons. Hence, there was a limit to the scenarios that could be presented. From an ethical point of view, the mystery client methodology is most feasible where physiological symptoms are not required to be present [28]. Consequently, the scenarios we used in this study excluded such. Also, as nursing students, the MCs might have been more confident in their interaction with health workers than the typical AYP. In this case, we traded off between having competent MCs and demonstrating as much as possible all the features of a typical AYP. In any case, it is worth noting that MCs are generally more likely to be bolder in their encounters than the typical AYP since they were selected and trained, and know that they are only merely acting. Finally, there might be some recall bias on the part of the MCs, but we tried to minimize this by ensuring that the post-visit interviews were conducted as soon as possible after the encounter, and compulsorily on the same day of the visit.

## Conclusion

The validation of the Adolescent Health Quality of Care (AHQOC) Index as a tool for evaluating the quality of adolescent sexual and reproductive health (ASRH) services is an important step towards improving the quality of care provided by healthcare facilities. The use of mystery clients in evaluating the quality of care provided by healthcare facilities has been increasingly recognized as a useful and effective approach. In this study, a 27-item tool was validated to allow for replication of methods in future studies and comparison of findings with other studies that adopt the index to assess ASRH services. This is a significant contribution to the field, as the reliability of study tools was something that had not been addressed in previous ASRH mystery client studies that used quantitative methods.

To improve ASRH services in low- and middle-income countries, including Nigeria, policymakers and healthcare providers should use the AHQOC Index as a benchmarking tool. The AHQOC Index can also be used to monitor the quality of care provided by healthcare facilities over time, which is crucial for ensuring that improvements are sustained. Future studies should use larger sample sizes and diverse populations to enhance external validity. This will allow for a more accurate assessment of the quality of care provided by healthcare facilities, as well as a better understanding of the factors that influence the quality of care.

Finally, the AHQOC Index can complement the Nigerian *National Standard and Minimum Package for Adolescent Healthcare* and serve as a means of evaluating facility-based ASRH interventions. By using the AHQOC Index, policymakers and healthcare providers can ensure that their interventions are evidence-based and effective in improving the quality of ASRH services. Overall, the validation of the AHQOC Index is a significant step towards improving the quality of ASRH services in low- and middle-income countries, and should be embraced by policymakers and healthcare providers as a tool for improving adolescent health outcomes.

## Supporting information

S1 File AHQOC index and scoring. (DOCX)

S1 Data AHQOC index for mystery client studies dataset. (DTA)
