# Supplementary material for: Validation of the Adolescent Health Quality of Care (AHQOC) index for mystery client studies
Source: PLoS One. 2023 Jun 15;18(6):e0285888. doi: 10.1371/journal.pone.0285888 (PMC10270626; doi:10.1371/journal.pone.0285888)
Supplement: S1 File — (DOCX) [file pone.0285888.s001.docx]

**Validation of the Adolescent Health Quality of Care (AHQOC) Index for Mystery Client Studies**

Olujide Arije, Jason Madan, Tintswalo Hlungwani

**AHQOC Index and Scoring**

***Instruction:*** *This debriefing questionnaire is to be administered to the mystery clients by a supervisor following each encounter at a health facility before visiting the next assigned health facility.*

| **AHQOC Items** | | **RESPONSE** |
| --- | --- | --- |
| **Accessibility**: Young people in the catchment area of the health facility are aware of the services it provides, find the health facility easy to reach and obtain services from it | |  |
|  | Did you find the facility easily? | (1) Yes  (0) No |
|  | Were there any directional signs outside the facility? | (1) Yes  (0) No |
|  | Were there any directional signs within the facility? | (1) Yes  (0) No |
| **Acceptable services:** Young people find the environment, setting, and procedures of health facilities appealing and acceptable | |  |
|  | Was the outside of the facility clean? | (1) Unclean  (2) Somewhat clean  (3) Very clean |
|  | Was the inside of the facility clean? | (1) Unclean  (2) Somewhat clean  (3) Very clean |
|  | Was there a separate waiting room for adolescents? | (1) Yes  (0) No |
|  | Were there posters on STDs and other SRH issues in the facility? | (1) Yes  (0) No |
|  | How long did you have to wait before being attended to? | (1) >60min  (2) 31-60min  (3) 16-30min  (4) ≤15min |
|  | How did you feel about the waiting time? | (1) Just OK  (0) Too long |
| **Competencies and motivation of health workers:** Service providers are skilled and motivated to provide health services to young people in adolescent/youth-friendly manner | |  |
|  | Was your medical history taken? | (1) Yes  (0) No |
|  | Was your social record taken? | (1) Yes  (0) No |
|  | Was your reproductive history taken? | (1) Yes  (0) No |
|  | Was your sexual history taken? | (1) Yes  (0) No |
|  | Were you asked about previous contraceptive use experience | (1) Yes  (0) No |
| **Privacy and confidentiality:** Service providers are sensitive to the needs of young people, and maintain their privacy and confidentiality in service provision | |  |
|  | Were you assured of confidentiality? | (1) Yes  (0) No |
|  | Were you counseled in a place where visual privacy was guaranteed? | (1) Yes  (0) No |
|  | Were you counseled in a place where auditory privacy was guaranteed? | (1) Yes  (0) No |
| **Appropriate package & effective services:** The services provided by health facilities to young people are evidence-informed and effective and in line with the nationally defined package (including health education | |  |
|  | Were you counseled on pregnancy prevention? | (1) Yes  (0) No |
|  | Were you counseled on any contraceptive methods? | (1) Yes  (0) No |
|  | Did you feel the provider had adequate time for you during consultation? | (1) Yes  (0) No |
|  | Did the provider give you an opportunity to ask questions? | (1) Yes  (0) No |
| **Global assessment scale** | |  |
|  | What did you think about the cost? | (1) Expensive  (2) Affordable  (3) No cost |
|  | In general, how did you find the counseling? | (1) Satisfactory  (0) Not Satisfactory |
|  | Will you recommend this facility to any of your colleague? | (1) Yes  (0) No |
|  | Did the health appear knowledgeable about the case you presented | (1) Yes  (0) No |
|  | Did he/she address your worries seriously | (1) Yes  (0) No |
|  | Did he/she explain to you adequately | (1) Yes  (0) No |
